# Detection of Inferior Vena Cava Thrombosis Extending into the Right Atrium Using Point-of-care Ultrasound

**DOI:** 10.5811/cpcem.2019.1.41041

**Published:** 2019-01-23

**Authors:** Justin Yanuck, Ghadi Ghanem, Shadi Lahham

**Affiliations:** University of California, Irvine, Department of Emergency Medicine, Irvine, California

## CASE PRESENTATION

A 74-year-old male with a history of metastatic prostate cancer presented to the emergency department with hypotension and shortness of breath. We assessed volume status using point-of-care ultrasound (POCUS) with a phased array probe in the subxiphoid orientation. This revealed a large inferior vena cava (IVC) thrombus extending from above the IVC bifurcation into the right atrium (Image, Video). The patient was started on intravenous heparin and fluids. Computed tomography (CT) pulmonary angiogram revealed an occlusive pulmonary embolism (PE) in the right lower lobe.

## DISCUSSION

Venous thromboembolisms are estimated to occur in 0.1% of patients; 1.5% of patients hospitalized with deep vein thrombosis (DVT) were diagnosed with vena cava thrombosis, of whom 12% had a pulmonary embolism.^1^,^2^ The mortality rate for IVC thrombosis patients is nearly double that of DVT patients.^1^,^3^ These patients can present with lower limb swelling or pain, lower back pain, fever, or elevated inflammatory markers.^3^ CT or magnetic resonance imaging is often used to make the diagnosis.^4^,^5^

Recently, ultrasound has shown promise for quick identification of IVC thrombus.^6^ The rapid ultrasound for shock and hypotension (RUSH) protocol, which incorporates assessment of IVC intravascular volume status, can be used for these undifferentiated patients to diagnose conditions not apparent with the standard physical exam.^7^,^8^ The RUSH examination led to the definitive diagnosis and etiology of this patient’s hypotension and dyspnea.

Once diagnosed, confirmation using CT imaging and admission to the hospital for anticoagulation and hemodynamic monitoring is recommended.^9^ Invasive treatments include angioplasty or local thrombolysis.^3^

Our case highlights the use of POCUS to quickly identify etiologies of hypotensive and dyspneic patients. Further imaging should be obtained in IVC thrombosis patients to rule out PE or additional clots.

**CPC-EM Capsule**

What do we already know about this clinical entity?

*Inferior vena cava thrombosis is a rare, life-threatening condition that usually requires advanced imaging techniques to diagnose*.

What is the major impact of the image(s)?

*This image highlights the utility of the rapid ultrasound for shock and hypotension protocol for finding both obvious and less-obvious pathologies*.

How might this improve emergency medicine practice?

*Rapid diagnosis of undifferentiated hypotensive patients using point-of-care ultrasound expedites care and medical intervention*.

## Supplementary Information

Video Rapid ultrasound for shock and hypotension protocol using the phased array probe in the subxiphoid orientation revealed a large thrombus situated in the inferior vena cava.

## Figures and Tables

**Image:**
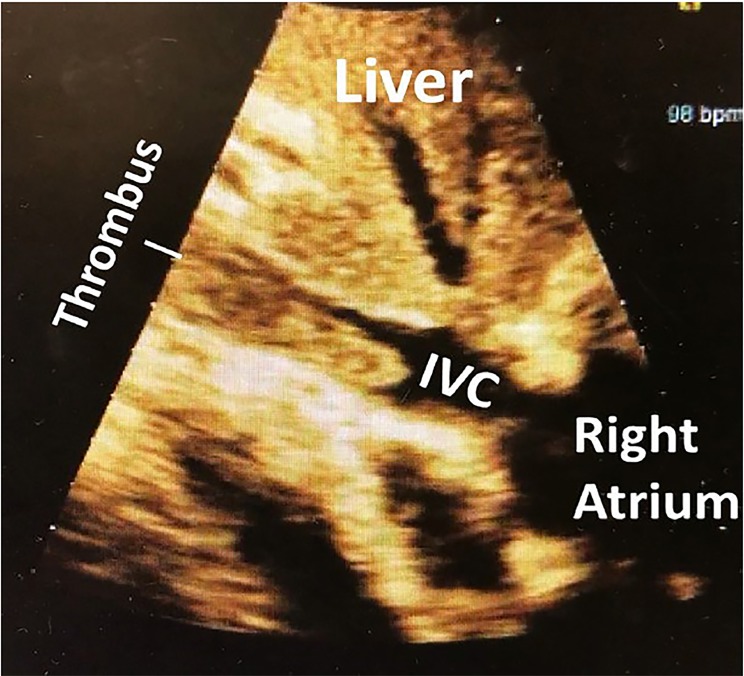
Point-of-care ultrasound phased array probe in the subxiphoid orientation revealed a thrombus situated in the inferior vena cava (IVC).
